# Accuracy of Predicting Residual Disease and Disease Progression During Active Surveillance for Esophageal Cancer

**DOI:** 10.1245/s10434-025-18531-y

**Published:** 2025-10-23

**Authors:** Sanjiv S. G. Gangaram Panday, David van Klaveren, Sjoerd M. Lagarde, Hester F. Lingsma, Bianca Mostert, Peter-Paul L. O. Coene, Jan Willem T. Dekker, Henk H. Hartgrink, Joos Heisterkamp, Merlijn Hutteman, Ewout A. Kouwenhoven, Grard A. P. Nieuwenhuijzen, Jean-Pierre Pierie, Johanna W. van Sandick, Meindert N. Sosef, Edwin S. van der Zaag, J. Jan B. van Lanschot, Bas P. L. Wijnhoven, SANO Study Group

**Affiliations:** 1 Department of Surgery, Erasmus MC Cancer Institute, University Medical Centre, Rotterdam, The Netherlands; 2 Department of Public Health, Erasmus MC, University Medical Centre, Rotterdam, The Netherlands; 3 Department of Medical Oncology, Erasmus MC Cancer Institute, University Medical Centre, Rotterdam, The Netherlands; 4 Department of Surgery, Maasstad Hospital, Rotterdam, The Netherlands; 5 Department of Surgery, Reinier de Graaf Gasthuis, Delft, The Netherlands; 6 Department of Surgery, Leiden University Medical Centre, Leiden, The Netherlands; 7 Department of Surgery, Elisabeth Tweesteden Hospital, Tilburg, The Netherlands; 8 Department of Surgery, Radboud University Medical Centre, Nijmegen, The Netherlands; 9 Department of Surgery, ZGT Hospital, Almelo, The Netherlands; 10 Department of Surgery, Catharina Hospital, Eindhoven, The Netherlands; 11 Department of Surgery, Medical Centre Leeuwarden, Leeuwarden, The Netherlands; 12 Department of Surgical Oncology, The Netherlands Cancer Institute, Amsterdam, The Netherlands; 13 Department of Surgery, Zuyderland Medical Centre, Heerlen, The Netherlands; 14 Department of Surgery, Gelre Hospital, Apeldoorn, The Netherlands

## Abstract

**Background:**

To date, active surveillance has been non-inferior to standard surgery for patients with esophageal cancer, achieving a clinical complete response (CCR) after neoadjuvant chemoradiotherapy (nCRT). However, two thirds of patients have residual disease detected 12 weeks after nCRT and undergo surgery. At 12 weeks, nearly half of the patients with CCR will experience locoregional regrowth. This study aimed to identify routine predictive factors for achieving (sustained) CCR to improve patient selection for active surveillance.

**Methods:**

Data from the SANO trial were analyzed, including data of patients who underwent nCRT for esophageal cancer. Logistic regression assessed predictors of CCR at 12 weeks, with potential factors including age, sex, WHO performance status, clinical T and N categories, histology, differentiation grade, tumor location, and tumor length. For patients with CCR in active surveillance, cause-specific proportional hazards regression identified predictors of sustained CCR (no locoregional regrowth, dissemination, or death) during a minimum 3-year follow-up period. Discrimination was quantified using the concordance statistic (c-statistic) with bootstrap validation.

**Results:**

Of 750 patients, 274 (37 %) achieved CCR at 12 weeks. Higher cN category was associated with lower likelihood of CCR (cN2–3 vs cN0: odds ratio [OR], 0.57; 95 % confidence interval [CI], 0.37–0.88; *P* < 0.01; c-statistic, 0.56). Among 198 patients in active surveillance, 25 % had sustained CCR after a median follow-up period of 54 months (interquartile range [IQR],46–58 months). Higher cN category (cN2–3 vs cN0; HR, 2.08; 95 % CI, 1.25–3.48; *P* < 0.01) was associated with non-sustained CCR (c-statistic, 0.58).

**Conclusion:**

Standard clinical parameters poorly predict clinical response after nCRT. Additional predictive parameters and better diagnostic tests are needed to improve patient selection for active surveillance.

**Supplementary Information:**

The online version contains supplementary material available at 10.1245/s10434-025-18531-y.

Active surveillance is a potential treatment strategy for patients with esophageal or junctional cancer who have a complete clinical response (CCR) 12 weeks after neoadjuvant chemoradiotherapy (nCRT).^1^ Results from the recently published SANO trial show that one third of patients had a CCR 12 weeks after nCRT.

A CCR was defined as no evidence or suspicion of residual locoregional tumor or metastases, based on two sequential clinical response evaluations. For nearly half of the patients in the active surveillance group, locoregional regrowth developed, with half of these cases occurring within the first 3 months after follow-up evaluation (6 months after completion of nCRT).^1^ Furthermore, distant metastases developed in 10 % of the patients in the active surveillance group during the same period.

It is important to explore whether a poor response to nCRT can be predicted based on patient and tumor characteristics. This insight could enable selection of patients with residual tumor before clinical response evaluations after nCRT, minimizing their exposure to additional diagnostic tests and preventing delay of surgery. Unnecessary response evaluations and unmet expectations could be avoided if patients in active surveillance with a high risk for the development of locoregional regrowth or distant metastases can be predicted. Moreover, when patients with a high probability of experiencing early distant metastases (within 6 months after completion of nCRT) can be identified, the performance of a possibly non-beneficial esophagectomy could be reconsidered.

The Neores II trial suggested possible harm of delaying esophagectomy for patients with tumor regression grade (TRG) 4 residual tumors after nCRT.^2^ Although no significant difference in overall survival or distant dissemination rate was observed between the active surveillance and surgery groups in the SANO trial at 2 years, the long-term disease-free survival has to be awaited.^1^ Therefore, patients with a CCR undergoing active surveillance should be carefully selected.

Previous studies have investigated the predictive value of endoscopy and positron emission tomography with computed tomography (PET-CT) to detect locoregional residual disease, finding minor predictive value.^3,4^ Furthermore, the prediction of a pathologic complete response after nCRT has been studied.^5,6^ However, prediction of residual disease or regrowth within an active surveillance cohort has not been explored to date and could be used to optimize patient management strategies.

This study aimed to predict CCR 12 weeks after completion of nCRT and to predict sustained CCR during active surveillance to better identify patients who could benefit from active surveillance.

## Methods

### Patients

Between November 2017 and January 2021, 776 patients with adenosquamous or squamous cell carcinoma of the esophagus or gastro-esophageal junction who underwent nCRT according to the CROSS regimen were included in the multicenter cluster-randomized SANO trial comparing active surveillance with standard surgery for patients with a CCR.^7,8^ Clinical tumor staging was assessed using endoscopic ultrasonography or CT, and clinical nodal staging was based on endoscopic ultrasonography. If endoscopic ultrasonography was not available, CT was used for nodal staging.

The study excluded all patients who underwent a PET-CT scan at baseline, and the primary tumor was required to be fluorodeoxyglucose (FDG)-avid. Patients who experienced metastases or died before undergoing any study procedure, withdrew consent, requested surgery, or experienced a major protocol violation during the first 12 weeks after nCRT also were excluded from the study.

Patients were included in the active surveillance group of the SANO trial after attaining a complete clinical response 12 weeks after nCRT.^1^ The prospectively collected SANO database was used in this study. Because the current analysis used the data from the SANO trial, which had already received ethical approval from the medical ethics committee of Erasmus MC (MEC-2017-392), no additional ethical approval was required.

### Procedures

All the patients underwent two clinical response evaluations to detect residual locoregional tumor or interval metastasis after nCRT. The first clinical response evaluation (CRE-1) was an endoscopy with a minimum of four bite-on-bite biopsies from the primary tumor location 4 to 6 weeks after nCRT. Patients who did not show residual tumor were scheduled for a second response evaluation (CRE-2). This was performed 10 to 12 weeks after nCRT and consisted of a PET-CT scan, an endoscopy, and an endoscopic ultrasound (EUS) with fine-needle aspiration of suspected lymph nodes. When no residual tumor was diagnosed or clinically suspected at CRE-2 (i.e., a complete clinical response [CCR]), the patients were enrolled in active surveillance, starting 10 to 12 weeks after nCRT, or underwent immediate surgery.

For the purpose of the current study, only those patients who underwent active surveillance were further selected. Patients who could not undergo clinical response evaluations, died, or had a non-passable stenosis within the first 12 weeks after nCRT were considered non-CCR.

The patients in the active surveillance group underwent repeated clinical response evaluations, identical to CRE-2, at 6, 9, 12, 16, 20, 24, 30, 36, 48, and 60 months after completion of nCRT. Response evaluations were terminated if the patient withdrew consent, experienced locoregional regrowth or metastases, or died. The patients who refused active surveillance after CRE-2 but did not withdraw consent still were monitored and analyzed.

### Outcome

The association of patient and tumor characteristics at baseline with CCR 12 weeks after nCRT and sustained CCR (defined as the absence of locoregional regrowth, distant metastasis, or mortality) during a minimum follow-up period of 3 years for the last patient under active surveillance was assessed. Finally, the association of patient and tumor characteristics with distant progression-free survival, defined as the absence of distant metastases or mortality, was analyzed. The analysis of sustained CCR and distant progression-free survival also inherently included post-nCRT findings (PET-CT, endoscopy, and EUS) because selection for active surveillance (for CCR) was based on these assessments.

### Selection of Clinical Predictors

The number of predictive parameters to be tested was determined based on the cohort size and the number of events (at least 10 events per predictor variable) within the active surveillance group, leading to the selection of 10 predictive parameters. The selection of parameters was based on previously published literature regarding the prediction of pathologic complete response, as well as on additional variables with a hypothesized association with pathologic complete response.^5,6,9,10^ The following parameters were selected for identification of predictive value on CCR at CRE-2 and sustained CCR: sex, age at diagnosis, tumor histology, tumor differentiation grade, cT category, cN category, World Health Organization (WHO) performance status, tumor location, number of completed chemotherapy cycles, and tumor length. Radiotherapy dosage was not included because only a small group of patients (1 %) had a dose reduction.

Because of small group sizes, WHO classification, number of chemotherapy cycles, cT category, differentiation grade, and tumor location were dichotomized (Table 1). The highest cN categories were grouped (cN2–3). Histology classified as “other” (e.g., adenosquamous, undifferentiated, high-grade dysplasia) was categorized as missing due to the small size and heterogeneity of the group. Undetermined cT category, cN category and tumor differentiation grade also were categorized as missing.

**Table 1:** Baseline characteristics of all included patients

	Total^a^ (*n* = 750) *n* (%)	Active surveillance^a^ (*n* = 198) *n* (%)
*Gender*		
Female	139 (19)	42 (21)
Male	611 (82)	156(79)
Median age: years (IQR)	68 (62–73)	69 (63–74)
*Tumor histology*		
Adenocarcinoma	591 (79)	147 (74)
Squamous cell carcinoma	136 (18)	47 (24)
Other	23 (3)	4 (2)
*Tumor differentiation grade*		
G1	118 (16)	33 (17)
G2	341 (46)	97 (49)
G3	276 (37)	65 (33)
Gx	15 (2)	3 (2)
*Clinical T category*^*b*^		
cT1	4 (1)	2 (1)
cT2	153 (20)	47 (24)
cT3	560 (75)	145 (73)
cT4	16 (2)	3 (2)
cTx	17 (2)	1 (1)
*Clinical N category*^*b*^		
cN0	288 (38)	86 (43)
cN1	287 (38)	77 (39)
cN2	156 (21)	31 (16)
cN3	14 (2)	4 (2)
cNx	5 (1)	0 (0)
*WHO performance status*^*c*^		
0	451 (60)	133 (67)
1	206 (28)	54 (27)
2	5 (1)	2 (1)
3	1 (0)	1 (1)
Missing	87 (12)	8 (4)
*Tumor location*^*d*^		
Proximal esophagus	5 (1)	3 (2)
Middle esophagus	75 (10)	24 (12)
Distal esophagus	465 (62)	120 (61)
Esophagogastric junction	205 (27)	51 (26)
Mean tumor length^d^	4.77 ± 2.40	4.71 ± 2.40
Missing	77 (10)	11 (6)
*Chemotherapy cycles*^*e*^		
Full	686 (92)	180 (91)
<5	64 (9)	18 (9)

IQR, interquartile range

^a^Percentages can add up above 100 % due to rounding.

^b^cTNM staging is reported according to the 7th edition of the Union for International Cancer Control staging manual. T staging and clinical nodal staging are based on endoscopic ultrasonography or computed tomography (CT).

^c^World Health Organization (WHO) performance score assessed on a 5-point scale. A higher number indicates greater disability.

^d^Determined by means of endoscopy

^e^51 Patients received four cycles; 8 patients received three cycles; 3 patients received two cycles; and 2 patients received one cycle. Of these patients, 75 % had their treatment reduced due to toxicity.

### Statistical Analysis

Single imputation was performed using Multivariate Imputation by Chained Equations (MICE) given the low percentage of missing values.^11^ A multivariable logistic regression model was used to assess the associations of the aforementioned clinical predictors with CCR at 12 weeks. The potentially non-linear relationship between age and CCR at 12 weeks was modeled with restricted cubic splines with three knots.^12^ Predictions were derived from the model to determine a subset of patients with less than 20 % probability of CCR at 12 weeks. This 20 % cutoff was chosen based on the 23 % probability of CCR in patients with adenocarcinoma.^7^ The regression coefficients of the final model were transformed to odds ratios, and discrimination was assessed.

Discrimination pertains to the prediction model's capability to differentiate between patients who experience the event and those who do not. This is measured by the concordance statistic (c-statistic), which is equivalent to the area under the receiver operating characteristic curve (AUC) when the analysis involves binary outcomes.^13^ A c-statistic above 0.7 indicates good discriminatory ability, with values closer to 1 reflecting stronger model performance.^14^ To correct for optimism, bootstrapping with 1000 samples was performed.

Cause-specific proportional hazards regression was used to analyze the association of the aforementioned clinical predictors with sustained CCR.^15^ Time to the first event was defined as the date of CCR (endoscopy at CRE-2) to the date of locoregional regrowth, date of distant metastasis, date of death, or date of last follow-up visit. Due to the limited number of events per cause, we assumed the strength of predictors’ associations (regression coefficients) to be the same across all causes instead of estimating cause-specific predictor effects separately. Thus, although the model accounts for competing risks by focusing on the time to the first event, it does not explicitly model multiple or subsequent events per patient.

Performance of the model was quantified with a c-statistic and corrected for optimism by bootstrapping with 1000 samples. The same analysis was performed to examine the association of clinical predictors with distant progression-free survival. Time until the first event was defined as the date of CCR to the date of distant metastasis, date of death, or date of last follow-up visit. For all analyses, *P* values lower than 0.05 were considered statistically significant.

Data were analyzed using R version 4.2.2 (R: A Language and Environment for Statistical Computing; The R Foundation for Statistical Computing, Vienna, Austria), using the packages *mice*, *rms*, and *mstate*.

## Results

From the 776 patients in the SANO trial, the study excluded 9 patients who died or experienced metastases before undergoing any study procedure, 8 patients who withdrew consent, 6 patients who requested immediate surgery despite allocation to active surveillance, and 3 patients who had major protocol violations, resulting in 750 patients in the current study. The majority of the patient population was male (82 %) with a median age of 68 years (interquartile range [IQR], 62–73 years). Most of the tumors were adenocarcinoma (79 %), cT3 (75 %), or located in the distal esophagus (62 %). The majority of the patients (92 %) received all five cycles of chemotherapy (Table 1).

**Fig. 1:**
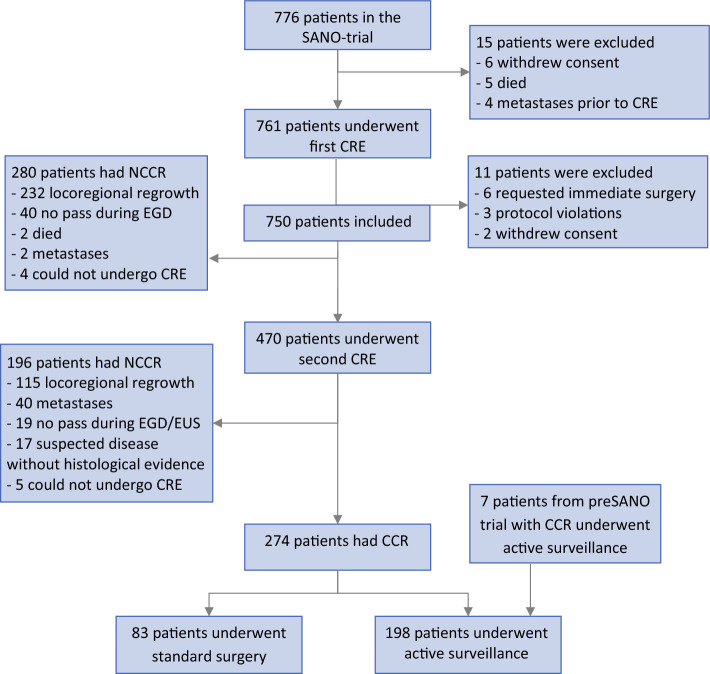
Patient flowchart. (N)CCR, (non)clinical complete response; CRE, clinical response evaluation; nCRT, neoadjuvant chemoradiotherapy; EGD, esophagogastroduodenoscopy; EUS, endoscopic ultrasound

Of the 750 patients, 274 (37 %) had a CCR 12 weeks after nCRT. Altogether, 198 patients underwent active surveillance (Fig. 1). After a median follow-up period of 54 months (IQR, 46–58 months), 49 of the 198 patients (25 %) had a sustained CCR, 99 (50 %) had a locoregional regrowth (49 [49 %] of whom experienced distant metastases), 37 (19 %) experienced distant metastases without locoregional regrowth, and 13 (7 %) died without disease recurrence.

Higher cN category was associated with a lower likelihood of initial CCR (cN2–3 vs cN0: odds ratio [OR], 0.57, 95 % confidence interval [CI], 0.37–0.87; *P* = 0.001; Table 2), based on 274 of 750 patients patients who achieved CCR. This was not observed for cN1 versus cN0. Among the patients with cN2–3 disease, 28.5 % achieved CCR. Other clinicopathologic parameters were not significantly associated. The initial c-statistic was 0.60, which dropped to 0.56 after correction for optimism, indicating poor discriminative ability. Only 0.3 % of the patients had less than 20 % predicted probability of CCR (Fig. S1).

**Table 2:** Associations between predictors and clinical complete response (CCR) in 750 patients who underwent nCRT

	Multivariable OR	95 % CI	*P* Value
*Gender*			
Male	Ref		
Female	1.01	0.66–1.53	0.960
*Age*			
per decade	1.19	1.00–1.43	0.070
Tumor histology			
Adenocarcinoma	Ref		
Squamous cell carcinoma	1.51	0.93–2.44	0.092
*Tumor differentiation grade*			
G1–G2	Ref		
G3	0.90	0.65–1.23	0.501
*cT category*			
cT1–cT2	Ref		
cT3–cT4	1.00	0.68–1.45	0.951
*cN category*			
cN0	Ref		
cN1	0.77	0.55–1.09	0.143
cN2–cN3	0.57	0.37–0.87	**0.001**
*WHO performance status*			
0	Ref		
Impaired	0.76	0.54–1.05	0.101
*Tumor location*			
Proximal-mid	Ref		
Distal junction	0.91	0.51–1.64	0.751
*Tumor length*			
per centimeter	1.00	0.93–1.07	0.981
*Chemotherapy cycles*			
Full	Ref		
<5	1.11	0.64–1.86	0.733

nCRT, neoadjuvant chemoradiotherapy; OR, odds ratio; CI, confidence interval; WHO, World Health Organization

Higher cN category also was associated with a lower likelihood of sustained CCR (cN2–3 vs cN0: hazard ratio (HR) for non CCR, 2.04; 95 % CI, 1.24–3.37; *P* = 0.005), based on 198 patients with initial CCR who entered active surveillance (Table 3). This was not observed for cN1 versus cN0. Among the patients with cN2–3 disease who achieved an initial CCR, 11.4 % experienced sustained CCR during the follow-up period. Other clinicopathologic parameters were not significantly associated. The c-statistic for this model was 0.60, which dropped to 0.56 after correction for optimism.

**Table 3:** Associations between predictors and sustained clinical complete response in 198 patients with CCR

	Multivariable HR^a^	95 % CI	*P* Value
*Gender*			
Male	Ref		
Female	0.83	0.53–1.29	0.407
*Age*			
per decade	1.09	0.88–1.36	0.439
*Tumor histology*			
Adenocarcinoma	Ref		
Squamous cell carcinoma	0.68	0.42–1.10	0.120
*Tumor differentiation grade*			
G1–G2	Ref		
G3	1.10	0.78–1.56	0.586
*cT category*			
cT1–cT2	Ref		
cT3–cT4	1.06	0.71–1.58	0.786
*cN category*			
cN0	Ref		
cN1	1.29	0.88–1.90	0.191
cN2–cN3	2.04	1.24–3.37	**0.005**
*WHO performance status*			
0	Ref		
Impaired	0.88	0.61–1.27	0.489
*Tumor location*			
Proximal-mid	Ref		
Distal-junction	1.08	0.57–2.06	0.805
*Tumor length*			
per centimeter	1.01	0.94–1.10	0.741
*Chemotherapy cycles*			
Full	Ref		
<5	1.02	0.57–1.82	0.953

CCR, clinical complete response; HR, hazard ratio; CI, confidence interval; WHO, World Health Organization

^a^HR is presented for non-CCR as sustained CCR is coded as 0 vs other events.

Squamous cell carcinoma was associated with a higher likelihood of distant progression-free survival (squamous cell carcinoma vs adenocarcinoma: HR, 0.51; 95 % CI, 0.27–0.94; *P* = 0.031), based on the 198 patients in active surveillance (Table 4). Among the patients with squamous cell carcinoma who achieved an initial CCR, 61.2 % remained metastasis- and death-free during the follow-up period. Other clinicopathologic parameters were not significantly associated. The c-statistic for this model was 0.63, which dropped to 0.58 after correction for optimism.

**Table 4:** Associations between predictors and distant progression-free survival in 198 patients with CCR

	Multivariable HR^a^	95 % CI	*P* Value
*Gender*			
Male	Ref		
Female	0.79	0.47–1.35	0.395
*Age*			
per decade	1.25	0.98–1.60	0.074
*Tumor histology*			
Adenocarcinoma	Ref		
Squamous cell carcinoma	0.51	0.27–0.94	**0**.**031**
*Tumor differentiation grade*			
G1–G2	Ref		
G3	1.03	0.69–1.54	0.876
*cT category*			
cT1–cT2	Ref		
cT3–cT4	1.02	0.64–1.62	0.947
*cN category*			
cN0	Ref		
cN1	1.58	1.00–2.48	0.050
cN2–cN3	1.69	0.95–3.01	0.075
*WHO performance status*			
0	Ref		
Impaired	0.93	0.61–1.43	0.745
*Tumor location*			
Proximal-mid	Ref		
Distal-junction	0.90	0.41–1.99	0.804
*Tumor length*			
per centimeter	1.05	0.96–1.14	0.301
*Chemotherapy cycles*			
Full	Ref		
<5	1.05	0.53–2.06	0.301

CCR, clinical complete response; HR, hazard ratio; CI, confidence interval; WHO, World Health Organization

^a^HR is presented for distant progression or death as no event is coded as 0 vs other events.

## Discussion

A higher cN category was associated with a lower likelihood of achieving and maintaining CCR up to a median of 54 months after nCRT. However, our models were considered insufficient to reliably predict CCR 12 weeks after nCRT or sustained CCR during active surveillance. A sub-analysis indicated that squamous cell carcinoma was associated with a higher likelihood of distant progression-free survival.

Various studies have reported on the development of prediction models for pathologic complete response (pCR) after nCRT for patients with esophageal cancer. The superior sensitivity of squamous cell carcinoma to nCRT, as evidenced by higher response rates compared with adenocarcinomas, is well known.^5,7^ Additionally, a higher cN category has been associated previously with lower response rates for patients with squamous cell carcinoma.^16^ However this association was not observed in two studies predominantly involving adenocarcinoma.^5,17^

Previous studies also have found associations between pCR and shorter tumor length (median, 2 cm [IQR, 1–5 cm] vs 3 cm [IQR, 2–7 cm]), low cT category, poor differentiation grade, and female sex.^5,16–18^ However, these factors were not associated with CCR in the current study. A prediction model on survival showed cN category to be an individual prognostic factor, with higher cN category correlating with lower survival rates.^19,20^ This underscores the importance of considering cN category in the prognostication and management of patients. In cases with uncertain eligibility for active surveillance, a high cN category may be decisive.

Although our results are not strongly discriminative on an individual level, they suggest that offering active surveillance to patients with cN2–3 disease should be approached with caution. In general, cN category is not adequately assessed, and improving this assessment could potentially influence treatment decisions, including the consideration of induction chemotherapy for patients with cN2–3 to more effectively manage the high risk of metastatic disease.^21^ Notably, when post-nCRT endoscopic findings are considered, they may outweigh patient and tumor characteristics in terms of predictive ability.^6,17^

Our objective was to predict CCR at 12 weeks without using post-nCRT findings, thereby potentially avoiding unnecessary diagnostic tests and delay of surgery. But our model was not able to select patients who may benefit from response evaluations and subsequent active surveillance. Furthermore, by focusing on patients enrolled in active surveillance, we also found that patient and tumor characteristics are not strongly associated with sustained CCR, and thus we could not identify individuals with a lower likelihood of maintaining CCR. This was despite our inclusion of post-nCRT findings of the PET-CT, endoscopy, and EUS because patients were selected for active surveillance based on these results. An exception may be patients with cN2–3 disease, who consistently showed worse outcomes, suggesting that they are at higher risk of non-sustained CCR despite the initial clinical response.

The relationship of patient and tumor characteristics with CCR has not been previously studied. Prediction of pCR based on pathologic examination of the resection specimen differs from CCR because residual tumors may be missed 12 weeks after nCRT given the false-negative rate of 23 % for CRE-1 and CRE-2. Therefore, patients with uncertain CCR also are classified as non CCR. However, predicting CCR is relevant because a large portion of this cohort underwent active surveillance and thus lacked pathologic staging within 4 months after nCRT. Baseline patient and tumor characteristics are unreliable predictors of pCR.^5^ Because we reached a similar conclusion for CCR, other biomarkers of response and optimization of diagnostic tests are needed.

For locoregional residual disease (i.e., primary tumor site and regional lymph nodes) detection, we rely on the endoscopy with bite-on-bite biopsies and EUS with fine-needle aspiration.^22^ After nCRT, most residual disease persists in the mucosa and submucosa.^23^ To increase sensitivity, intensified sampling should be considered.^24^ Valkema et al.^4^ showed that PET-CT scan has no added value in the detection of locoregional residual disease after nCRT due to its low specificity. Radiomics could potentially increase the accuracy of initial staging and restaging after nCRT, but models should undergo external validation and are unlikely to replace histopathologic evaluation.^25,26^ For detection of interval metastasis, PET-CT remains useful.^22^ Additionally, fibroblast-activation protein-inhibitor (FAPI) PET-CT may enhance the detection rate of residual disease and metastasis in esophageal cancer and could predict response to nCRT.^27^ Detection of circulating tumor DNA has been correlated with disease progression after chemoradiation and could be of clinical value for future response evaluations.^28,29^ Tumor genomic profiling in both esophageal squamous cell carcinoma and adenocarcinoma may further refine prediction of response to nCRT, but although a range of genetic alterations has been investigated, candidate markers often are subject to threshold effects and limited reproducibility, and most findings still await rigorous external validation.^30,31^

The limitations of this study should be addressed. Although we applied a competing risk framework by considering the first event in the analysis of sustained CCR, this approach does not capture multiple or sequential events for individual patients due to limited cause-specific events. Consequently, the model is not discriminative for combinations of events, such as locoregional regrowth followed by metastasis, and does not incorporate an expanded set of predictive covariates that might differentiate these outcomes. It may be hypothesized that the same clinical characteristics will be predictive for locoregional regrowth and metastases because many patients eventually experience metastasis after locoregional regrowth within active surveillance. However, the distant progression-free survival analysis showed an association with histology rather than the cN category. The wide confidence intervals suggest a high degree of uncertainty, making the findings less robust despite reaching statistical significance.

Importantly, despite these limitations, the study included a sufficient number of events to support adequate statistical power for the primary analyses. This suggests that the poor discriminative performance of the models was more likely due to the limited predictive value of standard clinical parameters rather than an insufficient sample size. It remains important to identify early metastases (i.e., within 6 months after nCRT) with more accurate diagnostics because it can be hypothesized that patients who experience early metastases after nCRT also benefit from an active surveillance strategy by avoiding non-beneficial esophagectomy. Another important limitation was that not all cN status was assessed by endoscopic ultrasonography, which could decrease the reliability of the cN category.

Finally, missing data for WHO performance status and tumor length as well as undetermined differentiation grade, cT category, and cN category were noted. The missing data for cT and cN categories were addressed by restaging with baseline CT, PET-CT, and EUS, resulting in only a small residual group of undetermined cases. This group, together with other missing data, was adequately imputed.

In conclusion, routine clinical and tumor parameters are insufficient for predicting clinical response after nCRT. Consequently, a need exists for exploration of new predictive parameters, such as molecular biomarkers and radiomics, and for improved diagnostic tests to enhance patient selection for active surveillance among esophageal cancer patients after nCRT. Until such improvements are made, active surveillance may be considered broadly for eligible patients, with the understanding that predicting the likelihood of avoiding surgery beforehand can be based only on overall probabilities and remains uncertain for the individual patient. An important exception may be patients with cN2–3 disease, who appear to have a consistently higher risk of residual or recurrent disease and may therefore be less suitable candidates for active surveillance.

## Supplementary Information

Below is the link to the electronic supplementary material.

Supplementary file1 (DOCX 52 kb)
