# Supplementary material for: Accuracy of Predicting Residual Disease and Disease Progression During Active Surveillance for Esophageal Cancer
Source: Ann Surg Oncol. 2025 Oct 23;33(2):946–54. doi: 10.1245/s10434-025-18531-y (PMC12765740; doi:10.1245/s10434-025-18531-y)
Supplement: Supplementary file 1 — Supplementary file1 (DOCX 52 kb) [file 10434_2025_18531_MOESM1_ESM.docx]

| **Table of content** | **Page** |
| --- | --- |
| Figure S1: Histogram of predictions for complete clinical response | 2 |
| Table S1: Members of the SANO study group | 3 |


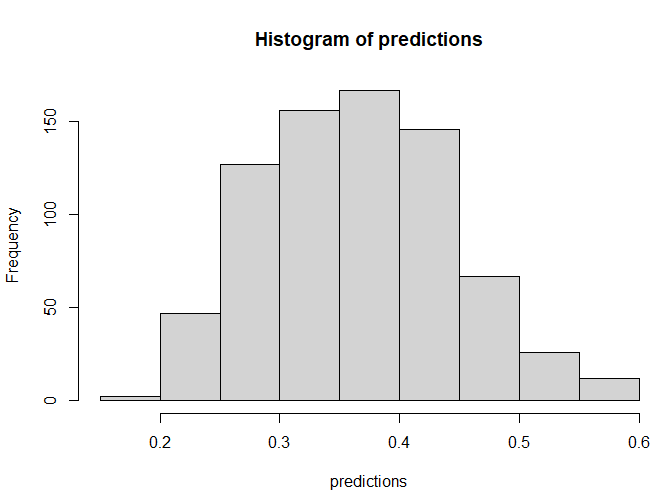


Figure S1 - Predictions of complete clinical response (CCR) for 750 patients who underwent nCRT X-axis: predicted probabilities of CCR , Y-axis: number of patients, corresponding to each range of predicted probabilities.

Table S1: Members of the SANO study group, besides the authors who are indicated on the title page

| Name | Department | Centre |
| --- | --- | --- |
| Manon C.W. Spaander | Gastroenterology | Erasmus MC – University medical centre |
| Arjun D. Koch | Gastroenterology | Erasmus MC – University medical centre |
| Suzan Nikkessen | Gastroenterology | Erasmus MC – University medical centre |
| Ate van der Gaast | Medical oncology | Erasmus MC – University medical centre |
| Roelf Valkema | Nuclear medicine | Erasmus MC – University medical centre |
| Michail Doukas | Pathology | Erasmus MC – University medical centre |
| Lindsey Oudijk | Pathology | Erasmus MC – University medical centre |
| Roy S. Dwarkasing | Radiology | Erasmus MC – University medical centre |
| Joost J. Nuyttens | Radiotherapy | Erasmus MC – University medical centre |
| Berend van der Wilk | Surgery | Erasmus MC – University medical centre |
| Ben Eyck | Surgery | Erasmus MC – University medical centre |
| Bo Jan Noordman | Surgery | Erasmus MC – University medical centre |
| Maartje Valkema | Surgery | Erasmus MC – University medical centre |
| Pieter C. van der Sluis | Surgery | Erasmus MC – University medical centre |
|  |  |  |
| Tanya M. Bisseling | Gastroenterology | Radboud University Medical Centre |
| Geert J. Bulte | Gastroenterology | Radboud University Medical Centre |
| Peter D. Siersema | Gastroenterology | Radboud University Medical Centre |
| Harm Westdorp | Medical oncology | Radboud University Medical Centre |
| Erik H. Aarntzen | Nuclear medicine | Radboud University Medical Centre |
| Chella S. van der Post | Pathology | Radboud University Medical Centre |
| Maartje C. van Rijk | Radiology | Radboud University Medical Centre |
| Pètra M. Braam | Radiotherapy | Radboud University Medical Centre |
| Heidi Rütten | Radiotherapy | Radboud University Medical Centre |
| Marcel Verheij | Radiotherapy | Radboud University Medical Centre |
| Camiel Rosman | Surgery | Radboud University Medical Centre |
| Bastiaan Klarenbeek | Surgery | Radboud University Medical Centre |
|  |  |  |
| Serge J. Zweers | Gastroenterology | Maasstad Hospital |
| Lieke Hol | Gastroenterology | Maasstad Hospital |
| I. Lisanne Holster | Gastroenterology | Maasstad Hospital |
| Ewout F.W. Courrech Staal | Radiology | Maasstad Hospital |
| Karen E. Hamoen | Pathology | Maasstad Hospital |
| Trudy Rapmund | Surgery | Maasstad Hospital |
| Erwin van der Harst | Surgery | Maasstad Hospital |
|  |  |  |
| Andries van der Linden | Gastroenterology | Ziekenhuisgroep Twente Hospital |
| Huseyin Aktas | Gastroenterology | Ziekenhuisgroep Twente Hospital |
| Polat Dura | Gastroenterology | Ziekenhuisgroep Twente Hospital |
| Ronald Hoekstra | Medical oncology | Ziekenhuisgroep Twente Hospital |
| Ali Agool | Nuclear medicine | Ziekenhuisgroep Twente Hospital |
| Joop van Baarlen | Pathology | Ziekenhuisgroep Twente Hospital |
| Ellen M. Hendriksen | Radiotherapy | Ziekenhuisgroep Twente Hospital |
| Henk Jan Mantel | Surgery | Ziekenhuisgroep Twente Hospital |
| Marc van Det | Surgery | Ziekenhuisgroep Twente Hospital |
|  |  |  |
| Rutger Quispel | Gastroenterology | Reinier de Graaf Gasthuis |
| Sana A. Mulder | Gastroenterology | Reinier de Graaf Gasthuis |
| Arjan J. Verschoor | Medical oncology | Reinier de Graaf Gasthuis |
| Marc R.J. ten Broek | Nuclear medicine | Reinier de Graaf Gasthuis |
| René J. Dallinga | Radiology | Reinier de Graaf Gasthuis |
| Karen J. Neelis | Radiotherapy | Reinier de Graaf Gasthuis |
| Erlinde de Graaf | Surgery | Reinier de Graaf Gasthuis |
| Stijn van Esser | Surgery | Reinier de Graaf Gasthuis |
|  |  |  |
| Jolanda M. van Dieren | Gastroenterology | The Netherlands Cancer Institute |
| Thomas R. de Wijkerslooth | Gastroenterology | The Netherlands Cancer Institute |
| Marieke A. Vollebergh | Medical oncology | The Netherlands Cancer Institute |
| Emilia C. Owers | Nuclear medicine | The Netherlands Cancer Institute |
| Annemarieke Bartels-Rutten | Radiology | The Netherlands Cancer Institute |
| Liudmila L. Kodach | Pathology | The Netherlands Cancer Institute |
| Francine E.M. Voncken | Radiotherapy | The Netherlands Cancer Institute |
| Yvonne Hilhorst | Surgery and gastroenterology | The Netherlands Cancer Institute |
| Marjolein Warmerdam | Surgery and gastroenterology | The Netherlands Cancer Institute |
|  |  |  |
| Sietske Corporaal | Gastroenterology | Medical Centre Leeuwarden |
| Edward Fiets | Medical oncology | Medical Centre Leeuwarden |
| Marco B. Polée | Medical oncology | Medical Centre Leeuwarden |
| Anne Marij G. van Burg | Nuclear medicine | Medical Centre Leeuwarden |
| Judith Nieken | Pathology | Medical Centre Leeuwarden |
| Rinze Wolf | Radiology | Medical Centre Leeuwarden |
| Vera Oppedijk | Radiotherapy | Medical Centre Leeuwarden |
| Marloes Emous | Surgery | Medical Centre Leeuwarden |
| Daniel A. Hess | Surgery | Medical Centre Leeuwarden |
|  |  |  |
| Eva Kouw | Gastroenterology | Gelre Hospital |
| Willemien Erkelens | Gastroenterology | Gelre Hospital |
| S. Cathrien S. Tromp – van Driel | Medical oncology | Gelre Hospital |
| Marc D. Zuijdwijk | Nuclear medicine | Gelre Hospital |
| H. Doornewaard | Pathology | Gelre Hospital |
| Karin Muller | Radiotherapy | Gelre Hospital, Radiotherapiegroep Deventer |
| Peter van Duijvendijk | Surgery | Gelre Hospital |
| Eelco B. Wassenaar | Surgery | Gelre Hospital |
|  |  |  |
| Wouter L. Curvers | Gastroenterology | Catharina Hospital |
| Geert-Jan Creemers | Medical oncology | Catharina Hospital |
| Mark J. Roef | Nuclear medicine | Catharina Hospital |
| Ineke van Lijnschoten | Pathology | Catharina Hospital |
| Joost Nederend | Radiology | Catharina Hospital |
| Maurice J.C. van der Sangen | Radiotherapy | Catharina Hospital |
| Tom C.G. Budiharto | Radiotherapy | Catharina Hospital |
| Fanny F.B.M. Heesakkers | Surgery | Catharina Hospital |
| Misha D. Luyer | Surgery | Catharina Hospital |
|  |  |  |
| Liekele E. Oostenbrug | Gastroenterology | Zuyderland Medical Centre |
| Fabienne A.R.M. Warmerdam | Medical oncology | Zuyderland Medical Centre |
| Wendy Schreurs | Nuclear medicine | Zuyderland Medical Centre |
| Bart de Vries | Pathology | Zuyderland Medical Centre |
| Roy F.A. Vliegen | Radiology | Zuyderland Medical Centre |
| Jeroen Buijsen | Radiotherapy | Zuyderland Medical Centre |
| Ilse Stohr | Surgery | Zuyderland Medical Centre |
| Eric H.J. Belgers | Surgery | Zuyderland Medical Centre |
|  |  |  |
| Jolein van der Kraan | Gastroenterology | Leiden University Medical Centre |
| Marije Slingerland | Medical oncology | Leiden University Medical Centre |
| Richard Raghoo | Nuclear medicine | Leiden University Medical Centre |
| A. Stijn L.P. Crobach | Pathology | Leiden University Medical Centre |
| Aart J. van der Molen | Radiology | Leiden University Medical Centre |
| Susan J.C.L.M. Quix | Surgery | Leiden University Medical Centre |
| Wobbe O. de Steur | Surgery | Leiden University Medical Centre |
|  |  |  |
| Wouter L. Hazen | Gastroenterology | Elisabeth Tweesteden Hospital |
| Laurens V. Beerepoot | Medical oncology | Elisabeth Tweesteden Hospital |
| David E. Ploeg | Pathology | Elisabeth Tweesteden Hospital |
| Tom Rozema | Radiotherapy | Elisabeth Tweesteden Hospital |
| Ilse A.C. Vermeltfoort | Nuclear medicine | Elisabeth Tweesteden Hospital |
| Walther Jansen | Surgery | Elisabeth Tweesteden Hospital |
